# Reporte de caso: Papel potencial de los corticosteroides en el tratamiento de la neumonía post-COVID-19

**DOI:** 10.1159/000521869

**Published:** 2022-01-24

**Authors:** Houari Aissaoui, Anaïs Eskenazi, Valentin Suteau, Antoine Adenis, Kinan Drak Alsibai

**Affiliations:** ^a^Departamento de Medicina, Unidad de Pulmonología, Centro Hospitalario Andrée Rosemon, Cayenne, Guayana; ^b^Departamento de Medicina, Centro Hospitalario Andrée Rosemon, Cayenne, Guayana; ^c^Departamento de Patología, Centro Hospitalario Andrée Rosemon, Cayenne, Guayana; ^d^Centre d'Investigation Clinique Antilles-Guyane (Inserm 1424), Centro Hospitalario Andrée Rosemon, Cayenne, Guayana; ^e^Centro de Recursos Biológicos (CRB Amazonie), Centro Hospitalario Andrée Rosemon, Cayenne, Guayana

## Abstract

Tras recuperarse de una neumonía grave debida a la enfermedad por coronavirus 2019 (COVID-19), algunos pacientes permanecen sintomáticos en el periodo posinfeccioso, ya sea de manera clínica, radiológica o respiratoria. El periodo post-COVID-19 se caracteriza por síntomas clínicos cuya duración varía de un sujeto a otro y no parece depender de la gravedad de la neumonía inicial. Las reacciones inflamatorias o inmunitarias persistentes en el periodo post-COVID-19 pueden desempeñar un papel en el desarrollo de las lesiones pulmonares. Aquí reportamos el caso de un hombre de 61 años con neumonía grave por COVID-19, complicada con síndrome de dificultad respiratoria aguda y embolia pulmonar, que requirió ingresar a la unidad de cuidados intensivos y oxigenoterapia de alto flujo. El paciente permaneció hospitalizado durante 23 días para tratar la neumonía grave por COVID-19. Posteriormente, se le envió a casa tras una prueba negativa de PCR para el SARS-CoV-2. El periodo posterior al COVID-19 se caracterizó por una sintomatología respiratoria compleja, con tos, disnea en reposo y disnea de esfuerzo que requirió oxigenoterapia durante varias semanas. Sorprendentemente, la tomografía computarizada torácica de seguimiento realizada cuatro semanas después del alta reveló lesiones pulmonares intersticiales bilaterales. Tras descartar una so­breinfección pulmonar, el paciente recibió tratamiento con corticosteroides orales durante tres meses en dosis digresiva. En nuestro caso, el uso de corticosteroides en la fase posterior al COVID-19 mejoró la enfermedad pulmonar. Estos beneficios se caracterizan por una rápida mejoría sintomática, la reparación acelerada de las imágenes pulmonares, la rápida retirada de oxígeno y un pronto retorno a las actividades cotidianas.

## Introducción

La enfermedad por coronavirus 2019 (coronavirus disease 2019, COVID-19) es una patología infecciosa pandémica causada por el nuevo coronavirus SARS-CoV-2 (severe acute respiratory syndrome coronavirus 2, coronavirus del síndrome respiratorio agudo severo 2). En la Guayana Francesa, el SARS-CoV-2 ha infectado a 15,664 habitantes y ha causado la muerte de 76 pacientes (según el informe epidemiológico de la Agencia Nacional de Salud de Guayana correspondiente a enero de 2021).

El diagnóstico final se basa en la detección del virus SARS-CoV-2 o en hallazgos por tomografía computarizada (TC) torácica típicos de neumonía por COVID-19, incluyendo opacidades en vidrio esmerilado e imágenes de consolidación, en su mayoría bilaterales, subpleurales y periféricas [1, 2].

Sin embargo, tras recuperarse de una neumonía grave por COVID-19, algunos pacientes permanecen sintomáticos en el periodo posinfeccioso, ya sea de forma clínica, radiológica o respiratoria, aunque la prueba de control del SARS-CoV-2 sea negativa [3, 4, 5]. El periodo post-COVID-19 se caracteriza por síntomas clínicos cuya duración varía de un sujeto a otro y no parece depender de la gravedad de la neumonía inicial [6]. La persistencia de la inflamación residual o de la respuesta inmunitaria, así como los mecanismos de reparación y remodelación en el periodo post-COVID-19 pueden desempeñar un papel en el desarrollo de nuevas lesiones pulmonares o en el agravamiento de lesiones preexistentes [7]. En este artículo, discutiremos el papel potencial del tratamiento con corticosteroides en la evolución favorable de la neumonía post-COVID-19. El paciente recibió tratamiento para una forma grave de neumonía por COVID-19, pero persistió la sintomatología respiratoria posinfecciosa, con imágenes radiológicas que favorecían una enfermedad pulmonar intersticial bilateral de progresión rápida.

## Reporte de caso

Reportamos el caso de un hombre de 61 años, no-fumador, con antecedentes de obesidad, asma intermitente bien controlada y discreta dilatación bronquial (DB) de causa indeterminada localizada en la língula, con antecedentes familiares de DB.

**Fig. 1:**
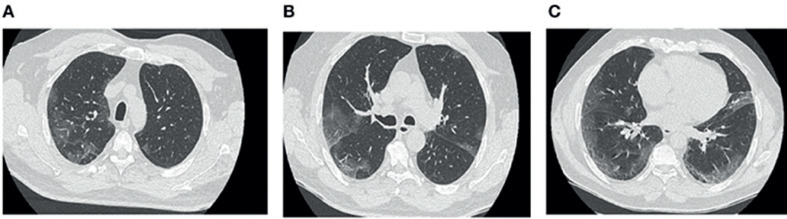
TC torácica de neumonía por COVID-19 (primera TC torácica). Los cortes axiales de la TC torácica a diferentes niveles muestran opacidades bilaterales en vidrio esmerilado e imágenes de consolidación bilaterales, subpleurales y localizadas principalmente en regiones posteriores (**A–C**).

El paciente acudió al servicio de urgencias de nuestro hospital en junio de 2020 tras la aparición de disnea y sibilancia difusa. Los síntomas comenzaron cuatro días antes con fiebre, tos seca, dolor torácico y diarrea. La TC torácica realizada al quinto día del inicio de los síntomas mostró opacidades en vidrio esmerilado sugestivas de COVID-19, con afectación pulmonar moderada (Figura 1). Este diagnóstico se confirmó mediante una prueba de RT-PCR positiva para SARS-CoV-2.

Debido a la levedad de los síntomas en esta fase, el paciente fue enviado a casa para su confinamiento. Sin embargo, volvió al servicio de urgencias dos días después, al agravarse la disnea. En ese momento, la desaturación era de 89% en el aire ambiental. Tras esta evolución, el paciente ingresó en la unidad COVID-19. Durante la hospitalización, el paciente desarrolló síndrome de dificultad respiratoria aguda (SDRA), caracterizado por un marcado agravamiento de los síntomas respiratorios, con mayores necesidades de oxígeno, de 6 L/min al segundo día de hospitalización y hasta 15 L/min al día siguiente. Ante este rápido deterioro, el paciente recibió corticoides a corto plazo, con metilprednisolona intravenosa 100 mg al día, y el anticoagulante enoxaparina (0.4 ml) dos veces al día.

Tres días después, el paciente fue trasladado a la unidad de cuidados intensivos tras aumentar la polipnea y la astenia. Se prescribió oxigenoterapia mediante Optiflow con una fracción de oxígeno inspirado (FiO_2_) de 100% y un flujo de aire de 50 L/min por seis días. La angiotomografía computarizada mostró una embolia pulmonar segmentaria posterior y basal derecha y empeoramiento de la afectación pulmonar. Por tanto, se introdujo el anticoagulante enoxaparina a una dosis curativa de 6,000 U dos veces al día y 6 mg de dexametasona al día, durante diez días. Como resultado, se observó una mejora progresiva clínica y respiratoria, y el flujo de oxígeno se redujo a 6 L/min. Así, el paciente fue trasladado de nuevo a la unidad COVID-19 para su seguimiento. Sin embargo, desarrolló un cuadro de estrés y ansiedad y rechazó la oxigenoterapia, retirando sistemáticamente la cánula de oxígeno.

Tras una notable mejoría de los síntomas y una prueba de control RT-PCR del SARS-CoV-2 negativa, se dio de alta al paciente a los 23 días de la aparición de los síntomas, con oxigenoterapia a 6 L/min.

**Fig. 2:**
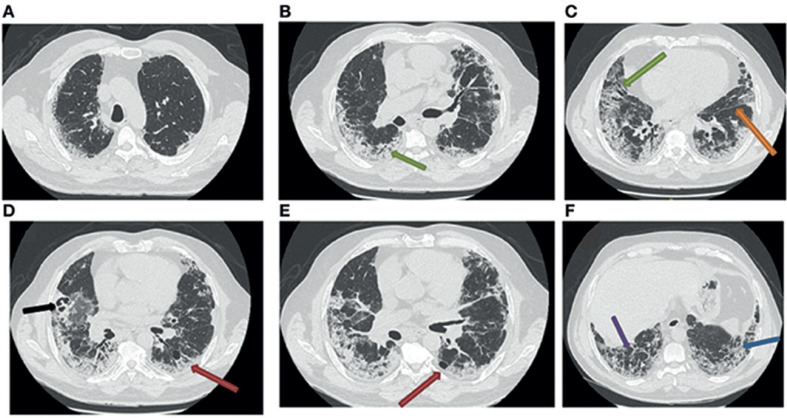
TC torácica de seguimiento de neumonía por COVID-19 (segunda TC torácica). La TC torácica realizada cuatro semanas después del tratamiento de la neumonía grave por COVID-19 revela la aparición de bronquiectasias (**A–C**; flecha verde), reticulaciones subpleurales (**F**; flecha azul), distorsiones lobulares (**F**; flecha violeta), deformación fisural (C; flecha naranja), enfisema paracicatricial (**D, E**; flecha roja) y cavitación en el lóbulo medio derecho (**D**; flecha negra).

Dos días más tarde, el paciente volvió al servicio de urgencias tras la reaparición de dificultad respiratoria durante su aseo matutino, con sensación de asfixia y disnea al hablar. La frecuencia respiratoria era 45 latidos/min y se registró una saturación de 87% con oxígeno a 6 L/min. La exploración funcional respiratoria (EFR) mostró un síndrome restrictivo grave, con una capacidad pulmonar total (CPT) de 39% del valor previsto. La TC torácica realizada cuatro semanas después del inicio de los síntomas reveló una lesión pulmonar intersticial difusa desarrollada sobre las imágenes del COVID-19 previamente observadas. La TC también mostró reticulación y distorsión globular, y bronquiectasias de tracción asociadas con opacidades en vidrio esmerilado y deformación fisural (Figuras 2A-F). También se observó un enfisema paracicatricial, sin signos de panal, y la aparición de una imagen de cavidad en el lóbulo medio derecho (Figuras 2D, E).

Tras las nuevas imágenes radiológicas, y para descartar una so­breinfección, se realizó una broncoscopia con lavado bronquioalveolar (LBA) en el lóbulo medio derecho, donde se localizaba la imagen de la cavidad (Figura 2D). El estudio citológico mostró una celularidad de 110,000 células/ml y una fórmula inflamatoria (macrófagos 76%, linfocitos 5%, neutrófilos 15%, y eosinófilos 4%), con pocos hematíes. El análisis bacteriológico y micológico del LBA por examen directo y cultivo no mostró la presencia de patógenos. El resultado de la prueba PCR para SARS-CoV-2 en el LBA fue negativo.

Ante esta inusual evolución radiológica, sugestiva de una enfermedad pulmonar intersticial de rápida aparición, se inició la corticoterapia con prednisolona (0.5 mg/kg/día) a finales de julio de 2020, con una reducción gradual de la dosis: 50 mg/día durante un mes, 40 mg/día por dos semanas, 30 mg/día durante dos semanas, 10 mg/día durante dos semanas, y finalmente 5 mg por dos semanas.

La evolución fue rápida y favorable tras el primer mes de corticoterapia con 0.5 mg/kg; los requerimientos de oxígeno disminuyeron a 4 L/min, y luego a 2 L/min, hasta completar el retiro a finales de agosto de 2020 (Tabla 1). Tras la fisioterapia motora establecida en el domicilio del paciente, la disnea de esfuerzo mejoró gradualmente. El paciente retomó sus actividades cotidianas privadas y profesionales a principios de septiembre de 2020.

**Fig. 3:**
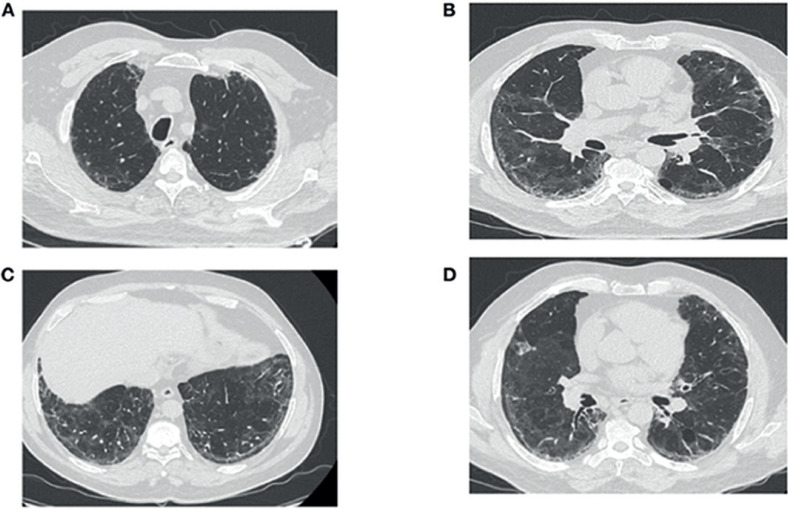
TC torácica posterior a la corticoterapia (tercera TC torácica). La TC torácica realizada luego de tres meses de corticoterapia para los síntomas posteriores al COVID-19 revela la regresión de las lesiones en vidrio esmerilado y las bronquiectasias, y la persistencia del enfisema paracicatricial (**A–C**). (**B–D**) Muestran la desaparición de la imagen de cavidad del lóbulo medio derecho, la regresión de los signos de retracción y la persistencia de pocas lesiones de reticulación.

La EFR en las visitas de seguimiento mostró que la CPT se normalizó al cuarto mes del inicio de la neumonía por COVID-19, que corresponde al tercer mes de corticoterapia (Tabla 2). Los análisis de sangre mejoraron gradualmente y volvieron a la normalidad a finales de octubre de 2020 (Tabla 3). Sorprendentemente, la tomografía computarizada torácica de seguimiento en el mismo periodo mostró una mejora significativa e inesperada de la lesión pulmonar, caracterizada por la desaparición de las bronquiectasias (Figuras 3A-C), una reducción importante de las reticulaciones y la regresión de los signos de retracción (Figuras 3B-D). En la TC también desapareció la imagen cavitaria del lóbulo medio derecho (Figura 3C), aunque persistió el enfisema paracicatricial (Figuras 3B, C).

## Discusión

En nuestro paciente, la afectación pulmonar relacionada con el SARS-CoV-2 fue extensa y grave, complicándose con SDRA y embolia pulmonar, que requirieron tratamiento en la unidad de cuidados intensivos con oxigenoterapia de alto flujo.

El periodo post-COVID-19 se caracterizó por una compleja sintomatología respiratoria, con tos, disnea en reposo y disnea de esfuerzo que requirió oxigenoterapia durante varias semanas; esto alteró la calidad de vida del paciente, quien sufrió estrés y ansiedad. Los síntomas se asociaron con el desarrollo de una enfermedad pulmonar intersticial difusa, grave y de progreso rápido, con signos de retracción, distorsión, cavitación y bronquiectasias difusas. La enfermedad pulmonar posterior al COVID-19 fue de rápida aparición, pues se produjo en las cuatro semanas siguientes a los primeros síntomas.

Estudios anteriores sobre la infección por SARS-CoV-2 demostraron que el daño pulmonar observado durante la fase inicial de la neumonía por COVID-19 es consecuencia de la respuesta inflamatoria e inmunitaria del huésped a la infección vírica. A nivel tisular, esta respuesta inflamatoria puede causar una lesión pulmonar difusa, con formación de membrana hialina, exudados de fibrina, daño epitelial e hiperplasia difusa de los neumocitos tipo II [8]. Otros análisis indican que los depósitos de fibrina intraalveolares e intersticiales y el infiltrado inflamatorio crónico pueden aparecer semanas después del diagnóstico de COVID-19 [9, 10]. En este contexto, cabe suponer que la respuesta inmunitaria extrema persiste en la etapa posterior a la infección por SARS-CoV-2 y sigue evolucionando durante varias semanas, lo que da lugar al establecimiento de un estado de inflamación crónica y daño pulmonar continuo.

En nuestro caso, la TC torácica de seguimiento realizada cuatro semanas después del alta reveló lesiones pulmonares intersticiales bilaterales, lo que sugiere una lesión inusual, similar a una fibrosis pulmonar de inicio rápido. Además, la TC mostró congestión vascular desarrollada sobre opacidades en vidrio esmerilado (Figuras 1A-C) y asociada con vasos marcadamente dilatados (Figuras 2B, E), lo que sugiere lesiones por microtrombosis. Recientemente se describieron estas lesiones similares a la fibrosis [11] y otros cambios vasculares pulmonares [12] relacionados con el COVID-19.

Estudios anteriores han sugerido que algunos pacientes con COVID-19 pueden desarrollar neumonía organizativa secundaria (NO) [13, 14, 15]. La NO se define como un exudado organizativo intraalveolar compuesto por fibroblastos y miofibroblastos [16]. La incidencia de NO fue de 12.5% en una cohorte alemana de pacientes con COVID-19 grave [13], y de 4% en un estudio observacional prospectivo más reciente [14]. Las infecciones virales son las etiologías más comunes de NO secundaria [17]. El diagnóstico final de NO requiere evaluación histológica mediante una biopsia [18].

Myall et al. utilizaron un patrón radiológico para definir la NO como la presencia de infiltrados subpleurales bilaterales en vidrio esmerilado asociados con una consolidación lineal densa subpleural y peribronquial, junto con bronquiectasias de tracción. Tras ofrecer a los pacientes tratamiento con corticosteroides, hubo mejoras en la función pulmonar y en las imágenes torácicas [14].

Nuestro paciente presentaba un patrón radiológico de NO con una consolidación irregular de distribución periférica y subpleural, asociada con reticulaciones subpleurales y bronquiectasias de tracción (Figuras 2B, D, E). Además, había enfisema paracicatricial y cavitación. En nuestro caso, la confirmación por biopsia no estaba indicada debido al estado del paciente.

Recientemente se describió la presencia de una cavitación en la neumonía por COVID-19 [19]. En presencia de dicha lesión, debe descartarse la sobreinfección. En nuestro caso, los resultados de los análisis citológicos, bacteriológicos y micológicos del LBA fueron negativos.

Los efectos del tratamiento con corticosteroides para reducir la mortalidad en casos críticos y graves de neumonía por COVID-19 están bien demostrados, con un nivel de evidencia intermedio [20].

**Fig. 4:**
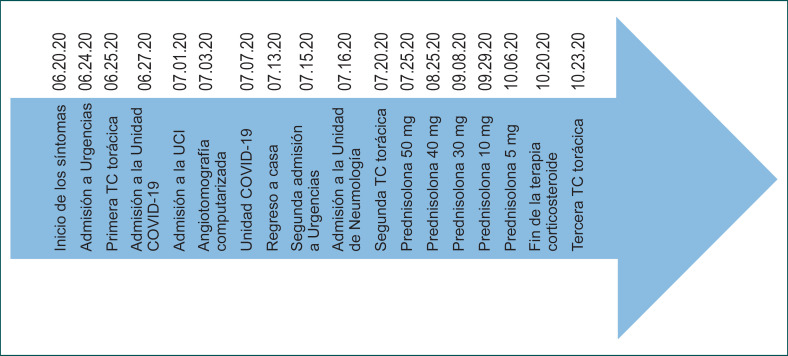
Esta línea de tiempo muestra el historial clínico del paciente desde el inicio de los síntomas relacionados con la neumonía por COVID-19 hasta la evolución favorable de los síntomas y las lesiones pulmonares tras el tratamiento con corticosteroides. Se dio de alta al paciente el 30 de julio de 2020 y se completó la terapia con corticosteroides en casa hasta el 20 de octubre de 2020.

En este reporte de caso, la terapia con corticosteroides en la etapa post-COVID-19 aceleró la reparación de las lesiones pulmonares, muy probablemente debido a sus efectos antiinflamatorios. Incluso se observó una reversión de las lesiones radiológicas tomadas inicialmente como signos de fibrosis pulmonar. El tratamiento con corticosteroides permitió también una rápida recuperación y normalización de la función respiratoria, así como la normalización de la presión parcial de oxígeno (PaO_2_) y un retiro completo del oxígeno tras sólo un mes de tratamiento (Figura 4, Tabla 1).

## Conclusión

Aquí reportamos un caso de lesión pulmonar grave por SARS-CoV-2 complicada con síndrome de dificultad respiratoria aguda y embolia pulmonar, que requirió ingreso en la unidad de cuidados intensivos y oxigenoterapia de alto flujo. El uso de la terapia con corticosteroides en nuestro paciente mejoró el curso de la enfermedad pulmonar. Observamos una rápida mejoría de los síntomas, una acelerada reparación de las imágenes radiológicas, un rápido retiro del oxígeno y una mejor tolerancia al ejercicio, con un rápido retorno a las actividades cotidianas.

La buena respuesta al tratamiento con corticosteroides en nuestro caso, que presentaba un patrón radiológico de NO, logró que el paciente volviera a su estado clínico inicial. Por tanto, creemos que el uso de corticosteroides es beneficioso en supervivientes de neumonía grave por COVID-19 que permanecen sintomáticos en el periodo posinfección y presentan características radiológicas consistentes con NO. No obstante, la eficacia de dicho tratamiento requiere validarse mediante ensayos aleatorios rigurosamente realizados, codificando la dosis y la duración del tratamiento.

## Declaración de disponibilidad de datos

Las contribuciones originales presentadas en el estudio se incluyen en el artículo/material suplementario; cualquier otra consulta puede dirigirse al autor corresponsal.

## Conflicto de interés

Los autores declaran que la investigación se llevó a cabo en ausencia de cualquier relación comercial o financiera que pudiera interpretarse como un potencial conflicto de interés.

## Información sobre licencias

Houari Aissaoui, Anaïs Eskenazi, Valentin Suteau, Antoine Adenis, Kinan Drak Alsibai: Case Report: Potential Role of Corticosteroids in the Management of Post-COVID-19 Pneumonia. Front Med (Lausanne). 2021;8:686806 (DOI: 10.3389/fmed.2021.686806). ^©^ 2021 Los Autores (traducción; contribuciones de los autores, declaración ética, nota del editor abreviadas), protegido por CC BY 4.0 (https://creativecommons.org/licenses/by/4.0/deed.es).

## Figures and Tables

**Tab 1:** Mejora gradual y consistente de la gasometría bajo tratamiento con corticosteroides de julio a octubre de 2020

	17 de julio	13 de agosto	27 de agosto	4 de septiembre	30 de octubre
pH (7.38–7.42)	7.45	7.43	7.41	7.42	7.42
PaO_2_ (75–100 mm Hg)	52	61	71	74	73
PaCO_2_ (38–42 mm Hg)	35	38	34	41	40
HCO_3_ (22–28 mEq/L)	24.3	25.2	21.6	26.6	25.9
SpO_2_ (94–100%)	90.0	94.1	96.0	96.9	95.3	

*pH, pH de la sangre arterial; PaO_2_, presión parcial de oxígeno; PaCO_2_, presión parcial de dióxido de carbono; SpO_2_, saturación de oxígeno; HCO_3_, bicarbonato.

**Tab 2:** Normalización de la función respiratoria tras tres meses de tratamiento con corticoides orales

	24 de julio	30 de octubre
CPT (%)	39	89.7
VR/CPT (%)	79.7	129.6
CVF (L)	1.78	2.81
CVF (%)	46	72.7
VEF_1_ (L)	1.37	2.30
VEF_1_ (%)	44.8	75.5
VEF_1_/CVF (%)	76.83	82	

VEF_1_, volumen espiratorio forzado en 1 s; CVF, capacidad vital forzada; CPT, capacidad pulmonar total; VR, volumen residual.

**Tab 3:** Los análisis sanguíneos mejoraron gradualmente y volvieron a la normalidad a finales de octubre de 2020

	17 de julio	4 de septiembre	24 de octubre
Hemoglobina (13–18 g/dL)	12.7	12.9	13.5
Plaquetas (150–400 g/L)	243	209	281
Fibrinógeno (2.38–4.98 g/L)	4.38	−	2.65
Proteína C reactiva (<5 mg/L)	41	0.8	0.5
Ferritina (20–200 µg/L)	429	−	220
Fosfatasa alcalina (40–130 U/L)	85	52	63
Dímero D (<500 ng/mL)	6206	2417	510
Deshidrogenasa láctica (135–225 U/L)	304	288	269	
